# Prophylactic effects of Bacille Calmette-Guérin intravesical instillation therapy: Time period-related comparison between Japan and Western countries

**DOI:** 10.1007/s11934-013-0374-1

**Published:** 2013-12-28

**Authors:** Takehiko Okamura, Ryosuke Ando, Hidetoshi Akita, Noriyasu Kawai, Keiichi Tozawa, Kenjiro Kohri, Hideo Arano

**Affiliations:** 1 Department of Urology, J.A. Aichi Anjo Kosei Hospital, 28 Higashihirokute, Anjo-cho, Anjo 446-8602 Japan; 2 Department of Nephro-Urology, Nagoya City University Graduate School of Medical Sciences, Nagoya, Japan; 3 Product Information Department, Japan BCG Laboratory, Tokyo, Japan

**Keywords:** Bacille Calmette-Guérin (BCG), Intravesical instillation, Non-muscle invasive bladder cancer (NMIBC), Prophylactic use, Time period-related comparison

## Abstract

Guidelines change every few years regarding the prophylactic use of Bacille Calmette-Guérin (BCG) against non-muscle invasive bladder cancer. We performed a retrospective comparison to clarify the differences in BCG efficacy, based on time period, between Japan and Western countries . Published literature on 18 Japanese and 28 Western patient studies were compared to evaluate differences in BCG efficacy. Additionally, Internet searches were performed to obtain comparative Japanese and Western data. BCG efficacy in Japanese literature tended to show decreasing non-recurrence rates by time period. Non-recurrence rates in Western countries increased each year. This discrepancy may stem from a number of factors, including changes in accepted BCG indications, the introduction of restaging transurethral resection (re-TUR), the concept of BCG maintenance, and the evolution of histopathological diagnostic criteria.

## Introduction

For the past two decades, intravesical Bacille Calmette-Guérin (BCG) instillation therapy has been considered the worldwide gold standard for the treatment of non-muscle invasive bladder cancer (NMIBC) [1–3]. Initially, there were no generally accepted guidelines or risk classifications. After trial and error, the first guidelines for BCG indication were published in 1999 by the American Urological Association (AUA) [1]. Subsequently, two major sets of guidelines were published by the European Association of Urology (EAU) [2] and the National Comprehensive Cancer Network (NCCN). These guidelines have been revised annually (http://www.nccn.org/professionals/physician_gls/PDF/bladder.pdf, http://www.uroweb.org/gls/pdf/05_TaT1_Bladder_Cancer_LR%20March%2013th%202012.pdf, http://www.auanet.org/content/clinical-practice-guidelines/clinical-guidelines.cfm?sub=bc). Additionally, several guidelines have been developed worldwide. In particular, guidelines for the prophylactic use of BCG against NMIBC have been revised due to modifications in the pathological diagnostic classifications, the introduction and consensus regarding restaging transurethral resection (re-TUR) procedures [3, 4, 5••], and other technical or mechanical improvements.

However, there have been no publications concerning the variations in outcome due to changes in the guidelines for BCG application. Therefore, we conducted a retrospective comparison to clarify differences in BCG efficacy, according to time period, between patients in Japan and Western countries.

## Methods

### Data Selection and Collection

#### Japanese Literature Records

A total of 18 papers (22 treatment arms) were chosen according to the criteria described below [6–23]. The literature searches were conducted to include reports published between January 1985 and July 2011. All literature was identified via PubMed database searches, which were limited to human subjects and contained the MeSH term “bladder neoplasms” with additional search terms “BCG” or “bacillus Calmette-Guérin” and “Japan.” Additional searches were conducted on the Japanese electronic database JAPIC (Japan Pharmaceutical Information Center). All identified studies were conducted within Japan. The beginning of the studies was clearly described, and the study registration periods occurred within five years. The enrolled cases were limited to those of intermediate to high risk, and the studies did not focus on particular patient categories such as those with carcinoma in situ (CIS) or T1G3 disease. Combinations with other agents that might be influenced by the effects of BCG were excluded, and other anticancer agents were not used.

#### Western Literature Records

The literature searches included reports published between January 1985 and May 2012. The searches were limited to reports of human subjects that were published in the English language and included the MeSH term “bladder neoplasms” with additional search terms “BCG” or “bacillus Calmette-Guérin.” The initial database search returned a collection of 1,833 reports, which were subsequently narrowed to 228 reports. From these reports, 28 reports (38 treatment arms) were chosen [24–50, 51••], according to the same conditions that were used to select Japanese patient literatures, which are described below. All studies were performed outside of Japan. The beginning of the studies was clearly described, and the registration periods occurred within 5 years. The enrolled cases were limited to those of intermediate to high risk, and the studies did not focus on particular patient categories such as those with CIS or T1G3 disease. Combinations with other agents that might be influenced by the effects of BCG were excluded, and other anticancer agents were not used.

A non-recurrence rate graph based on the above-described data was plotted for each time period at the beginning of the study. In studies that contained two or more BCG treatment arms, every arm was plotted.

#### Statistical Analyses

Simple linear regression analysis was used to determine the correlation coefficient between the period of BCG treatment and clinical variables. All statistical analyses were performed using Microsoft Office Excel 2007.

## Results

Most of the Japanese literature reports were published before 2000 [6–17]. Recent reports focused mainly on particular cases such as T1G3 cases or on specific institutional outcomes. BCG studies greater than 10 years in length were reported with increasing frequency after 2000; these reports were unsuitable for our study. Surprisingly, in the 1980s and early 1990s, patients with Ta, G1, or G2 cases were often overlooked [6–14].

This trend changed after a survey by the Japanese Urological Association in 1999–2001 [52]. The patient backgrounds that were considered widened dramatically, and BCG was used to treat many T1 and/or G3 cases. During the survey period, BCG was normally used for high-risk cases.

**Fig. 1:**
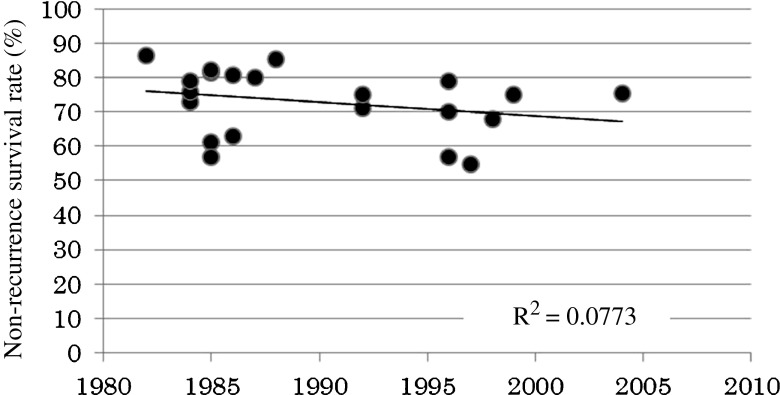
Analysis of the correlation between the year of BCG treatment initiation and the 3-year non-recurrent survival rate according to the 18 Japanese patient literature references

Figure 1 shows the initial treatment year of the BCG intravesical therapy studies and the 3-year non-recurrence rates. Although the 3-year non-recurrence rate appeared to have decreased gradually in recent years, there was no statistical correlation between the starting year and the 3-year non-recurrence rate.

**Fig. 2:**
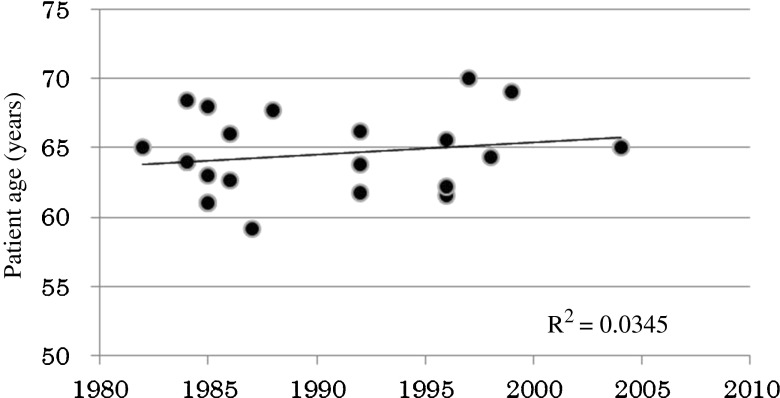
Median age distribution according to the 18 Japanese patient references

The correlation between treatment time period and patient age was examined. A trend toward increased patient age was seen in recent years, but no statistical correlation was observed (Fig. 2).

A summary comparison of literature data between the late-1990s and earlier periods demonstrates that BCG is widely used in high-risk patients such as T1G3 cases. Furthermore, it is possible that patient age has an effect on therapeutic outcome.

**Fig. 3:**
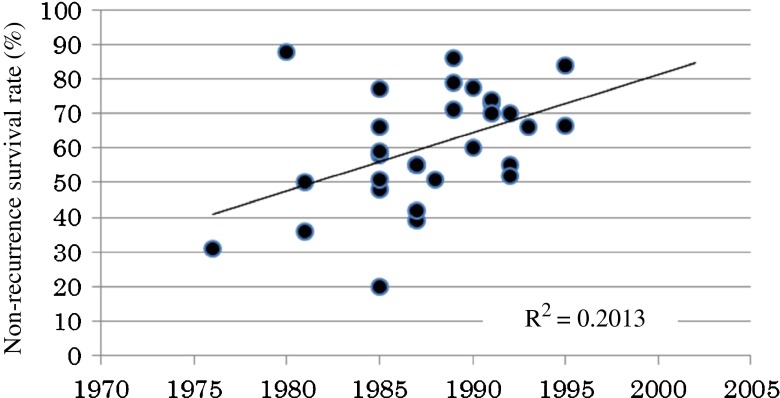
Analysis of the correlation between the year of BCG treatment initiation and the 3-year non-recurrence survival rate according to the 28 literature references of patients in Western countries

On the other hand, our analyses of the data from Western countries showed a trend toward increased non-recurrence rates (Fig. 3).

With regard to patient background, the percentage of high-risk patients has increased both in Japan and in Western countries (Europe and the United States).

**Fig. 4:**
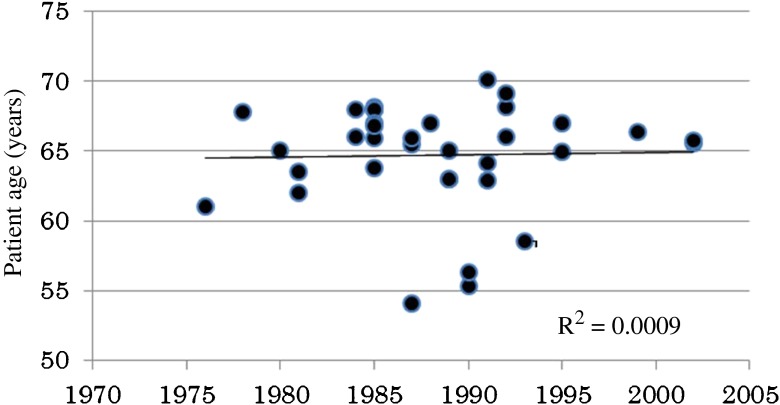
Median age distribution according to the 28 references of patients in Western countries

The reported median age of patients in Japanese literature increased by approximately 2 years over a period of greater than 20 years; however, the median age of patients in Western countries was almost unchanged over the time periods studied (Fig. 4).

## Discussion

The results of our study indicated a trend toward decreasing non-recurrence rates in Japanese patients after the year 2000, which may be related to differences in study background characteristics such as greater average numbers of tumors per case or increased numbers of low-dose cases. On the other hand, the prophylactic effects of BCG appeared to increase annually in Western countries. We believe that this is the first literature review of BCG intravesical instillation therapy stratified by age.

Clearly, there are a number of factors that may account for the trend in the Japanese cases. In the 1980s, BCG was introduced as a promising treatment option for recurrent NMIBC (initially referred to as superficial bladder cancer), which led to the global use of BCG. However, many severe adverse effects were reported, including anaphylactic shock, arthritis, Reiter’s syndrome, general tuberculosis infection, and contracted bladder. In response to this, applied doses were reduced [49, 53]. However, most dose-dependent comparisons did not show dose-related variations in efficacy [54, 55]. Additionally, the BCG indication criteria were changed so that more stringent patient selection guidelines were provided annually in the United States and other countries (AUA, EAU, and NCCN guidelines) (http://www.nccn.org/professionals/physician_gls/PDF/bladder.pdf, http://www.uroweb.org/gls/pdf/05_TaT1_Bladder_Cancer_LR%20March%2013th%202012.pdf, http://www.auanet.org/content/clinical-practice-guidelines/clinical-guidelines.cfm?sub=bc). The applied criteria were also changed in Japan (bladder cancer practice guideline: Japanese Urological Association, 2009).

The Spanish Urological Club for Oncological Treatment reported on a scoring system for factors that affected prognosis in patients who had received intravesical BCG therapy [56]. Age and gender were newly discovered prognostic factors in this study, in contrast to those reported by the European Organisation for Research and Treatment of Cancer, which accounted for almost no BCG cases. However, T-classification and tumor size were not found to be prognostic factors. These factors may have influenced BCG therapy outcomes in Western countries to some extent.

Although changes in histopathological bladder cancer grading and staging criteria could have an impact, the distinction between high-malignant potential and non-invasive low-malignant potential tumors has received more emphasis. Consequently, the name “superficial bladder cancer” was changed to NMIBC, and in Japan, pathological classifications were modified in the 1990s from T1a and T1b to T1 only.

Patient age is another potential factor in Japanese cases. In fact, a trend toward increased patient age at BCG application was observed in the 18 Japanese papers reviewed, as shown in Fig. 2. Several reports, including our own, have described lower efficacy rates for BCG therapy in elderly patients [57–59, 60••]. In Japanese literature, the median patient age increased by approximately 2 years over a 20-year period. Almost no change in patient age was observed in the Western countries (Fig. 4). The relationship between patient age and BCG efficacy is still not clearly understood.

Number of intravesical BCG instillations is another possible factor. Maintenance therapy has been established in Europe and the United States, and intravesical instillation time is therefore greater than in Japanese cases.

Initially, large numbers of BCG administrations were reported in the Japanese cases, but more recently, the number of administrations was reduced to 6 or 8. BCG for intravesical, with a treatment schedule of 8 administrations, was first approved in Japan in 1996. Maintenance administrations were approved in 2010.

We found no direct correlation between patient background and therapeutic strategy transitions. Trial-and-error variability, periods in which targets and methods were more firmly established, and periods of increased maintenance therapy were found to exist in all time periods

## Conclusions

The results of this study revealed an annual trend toward decreasing non-recurrence rates in Japanese studies. However, these results conflicted with those from studies in Western countries. This discrepancy may stem from a number of factors, including changes in accepted BCG indications, the introduction of re-TUR, the concept of BCG maintenance, and the evolution of histopathological diagnostic criteria.
